# Gaussian Elimination-Based Novel Canonical Correlation Analysis Method for EEG Motion Artifact Removal

**DOI:** 10.1155/2017/9674712

**Published:** 2017-10-08

**Authors:** Vandana Roy, Shailja Shukla, Piyush Kumar Shukla, Paresh Rawat

**Affiliations:** ^1^Department of Electronics and Communication, Jabalpur Engineering College, Jabalpur 482011, India; ^2^Department of Computer Sciences and Engineering, Jabalpur Engineering College, Jabalpur 482011, India; ^3^Department of Computer Sciences and Engineering, University Institute of Technology, Bhopal 462023, India; ^4^Department of Electronics and Communication, Truba Group of Institute, Bhopal 462023, India

## Abstract

The motion generated at the capturing time of electro-encephalography (EEG) signal leads to the artifacts, which may reduce the quality of obtained information. Existing artifact removal methods use canonical correlation analysis (CCA) for removing artifacts along with ensemble empirical mode decomposition (EEMD) and wavelet transform (WT). A new approach is proposed to further analyse and improve the filtering performance and reduce the filter computation time under highly noisy environment. This new approach of CCA is based on Gaussian elimination method which is used for calculating the correlation coefficients using backslash operation and is designed for EEG signal motion artifact removal. Gaussian elimination is used for solving linear equation to calculate Eigen values which reduces the computation cost of the CCA method. This novel proposed method is tested against currently available artifact removal techniques using EEMD-CCA and wavelet transform. The performance is tested on synthetic and real EEG signal data. The proposed artifact removal technique is evaluated using efficiency matrices such as del signal to noise ratio (DSNR), lambda (*λ*), root mean square error (RMSE), elapsed time, and ROC parameters. The results indicate suitablity of the proposed algorithm for use as a supplement to algorithms currently in use.

## 1. Introduction

EEG signal is widely used for exploring the human brain activity and is preferred over other physiological signals because they can be used to detect directly brain electrical activity changes over spans of millisecond time, whereas functional magnetic resonance imaging (fMRI) has time resolutions in seconds or minutes. Usually, EEG signal suffers from various motion artifacts generated at the capturing time. There are two main sources of artifact in neural signals other than the machine and environment. These are the muscular and ocular activities of the individual which generate low-amplitude, low-frequency electrical pulses that fall in filter range of sensors and recording equipment.

The EEG signal is contaminated by various artifacts as electrocardiogram (ECG), electrooculogram (EOG), and electromyogram (EMG). The EMG artifact is of more interest as it is having higher amplitude, broad spectrum, and variable topographical distribution than EEG signal [1]. The EMG artifacts have lower autocorrelation than EEG signal due to its wide frequency spectrum. Moreover, these artifacts resemble as temporal white noise. Therefore, artifact rejection is a fundamental research topic and is well researched in [2]. The EEMD is a data-driven and noise-assisted approach which is applied to remove motion artifacts from single-channel EEG signal [3].

A component-based automated separator of artifacts is required to linearly decompose the signals into source components. The components give the individual nature of information, where artifact information combines into separate sources and reconstruction of signals without these sources are claimed as artifact-free information. The performance of blind source separation (BSS) methods as independent component analysis (ICA) and CCA for EEG signal eye blink artifact removal is compared in [4] and concluded that CCA is more accurate and faster than ICA. The CCA algorithm performs better than ICA for muscle artifact removal because these artifacts are generated due to movement of body muscle group. Moreover, these artifacts do not represent stereotyped topography [5]. Anastasiadou et al. [6] applied CCA algorithm to remove muscle artifact from EEG signal. Moreover, artifact removal approach is improved by applying WT after CCA algorithm for automatic detection and removal of muscle artifact from EEG signal [7]. The cascaded combination of EEMD and CCA techniques is applied for single-channel EEG artifact removal in [8, 9]. The single-channel EEG signal is converted into the multidimensional signal by EEMD technique. In CCA, a second-order statistics is applied to segregate the artifact components from the input signal and its performance is compared and presented better in comparison of existing wavelet denoising and EEMD-ICA cascaded algorithms. Chen et al. [10] have improved the filtering approach by applying EEMD and MCCA (multiset CCA) to remove EMG with more computational time. To remove ocular artifacts automatically, the cascaded combination of CCA and WT algorithms is proposed in [11] and demonstrated that the proposed method removes artifacts significantly with preserving neural activity of the original signal. Safieddine et al. [12] suggested that EEMD algorithm outperforms over other stochastic and deterministic artifact removal approaches and WT algorithm performs well in the case of less noisy data.

An efficient cascaded approach EEMD-CCA-SWT is found successful for EEG motion artifact removal [13]. The combination of EEMD, CCA, and SWT approaches has been applied for effective suppression of the motion artifact from EEG signal. This three-stage cascaded approach removes the artifacts effectively with increased computational cost. This computational complexity is reduced in this research paper by developing an existing correlation-based algorithm with Gaussian elimination (GE) and inserted at the cascade of EEMD-SWT, leading to EEMD-GECCA-SWT which is the combination of EEMD and an improved approach GECCA (Gaussian elimination canonical correlation analysis) with SWT. This increased computational cost is effectively reduced due to applying GECCA approach in place of CCA. The left matrix division applied in GECCA allows better estimates for the matrix inversion; therefore, it improves the SWT filtering efficiency and thus improves the overall efficiency of the EEG motion artifact removal.

The paper organization is as the following. The artifact removal methods are recalled in Section 2, and then the proposed algorithm is discussed in Section 3. Details of applied EEG dataset is given in Section 4; results obtained by methods are presented, compared in tabular form, and detailed discussed in Section 5. Finally, conclusion is in Section 6.

## 2. Artifact Removal Methods

### 2.1. Ensemble Empirical Mode Decomposition

The EEMD algorithm decomposes a signal into a number of intrinsic mode functions (IMFs) through an iterative method termed as sifting [12]. At first level, the IMF1 is the mean of upper and lower envelop of original EEG signal *X*(*t*). Then residual signal is obtained by subtracting IMF1 from *X*(*t*). This process is iterated till stopping criterion is fulfilled (residual signal energy content is close to zero). The remaining residual signal is

(1) $$\begin{array}{c} P_{n}\left(t\right)=P_{n-1}\left(t\right)-{IMF}_{n}\left(t\right), \end{array}$$

where *P*_*n*_(*t*) = *X*(*t*).

Finally, the signal is reconstructed by adding all IMFs and residual signal as

(2) $$\begin{array}{c} X\left(t\right)=P_{n}\left(t\right)+\overset{N}{\underset{i=1}{\sum }}{IMF}_{i}\left(t\right). \end{array}$$

### 2.2. Gaussian Elimination Canonical Correlation Analysis (GECCA)

The standard existing CCA algorithm starts with an assumption that *X*[*n*] and *Y*[*n*] are two sets of random variables [4]. *X*[*n*] is the input vector of matrix and *Y*[*n*] is defined as temporally correlated by 2-D valid convolution operator from *X*[*n*] vectors using the linear convolution mask [1 0 1] as

(3) $$\begin{array}{c} Y\left[n\right]=conv2\left(X,\left[1\;0\;1\right]\right). \end{array}$$

Merging both input vectors as

(4) $$\begin{array}{c} Z=\left[X;Y\right]. \end{array}$$

If *ρ* is the maximum canonical correlation, *Cxx*, *Cyy* are auto covariances of vectors *X* and *Y*, respectively, and *Cxy* and *Cyx* are the cross covariance between vectors *X* and *Y*. The various possible correlation matrixes are

(5) $$\begin{array}{c} Cxx=C\left(1:sx,1:sx\right)+\beta *eye\left(sx\right), \end{array}$$

where *sx* is the size of the *X*, *sy* is the size of the *Y*, *β* is the predefined small residual constant which is set to 10^−8^ in this paper which is used by standard CCA method [2], and eye is the identity matrix with diagonal terms as 1 and all other terms are zero. The term *β*∗eye(*sx*) is used in order to set and maintain the initial nonzero value of the covariance matrix *Cxx*. The small value of *β* does not affect the covariance matrix values much.

(6) $$\begin{array}{c} Cxy=C\left(1:sx,sx+1:sx+sy\right),\\ Cyx=Cxy^{\prime },\\ Cyy=C\left(sx+1:sx+sy,sx+1:sx+sy\right)+ç*eye\left(sy\right),\\ {Cyy}^{-1}=inv\left(Cyy\right). \end{array}$$

Two different canonical solutions are obtained from *Z* by calculating the covariance as

(7) $$\begin{array}{c} C=\text{cov}\left(Z.^{\prime }\right). \end{array}$$

This is equivalent to two linear equations in *X* and *Y* vector directions, respectively, as

(8) $$\begin{array}{c} p\left[n\right]=a*X\left[n\right],\\ q\left[n\right]=b*Y\left[n\right], \end{array}$$

where *a* and *b* are weight vectors and *p*[*n*] and *q*[*n*] are canonical variates correspondent to *X*[*n*] and *Y*[*n*], respectively.

The maximum correlation between variables *a* and *b* are calculated as [8]

(9) $$\begin{array}{c} \text{max}\left(\rho \right)=\frac{a^{T}C_{xy}b}{\sqrt{a^{T}C_{xx}a}\sqrt{b^{T}C_{yy}b}}. \end{array}$$

Now, the demixing matrix *W* is calculated by simplifying (9) as

(10) $$\begin{array}{c} \rho^{2}a=inv\left(Cxx\right)*Cxy*inv\left(Cyy\right)*Cyx*a, \end{array}$$

(11) $$\begin{array}{c} \rho^{2}b=inv\left(Cxx\right)*\left(Cxy\right)*inv\left(Cyy\right)*Cyx*b, \end{array}$$

where *ρ*^2^ is calculated by obtaining Eigen values (*k*) of (10) and (11) and further, *a* and *b* variables are equal to Eigen vectors for the greatest value of *k* (Eigen value).

These weight vectors *a* and *b* are replaced in (8) to calculate canonical variates *p*[*n*] and *q*[*n*]. Moreover, the demixing matrix *W* can be calculated by inverse of estimated weight vector (*W* = *a*^−1^). Finally, the source *S*[*n*] is estimated by applying *W* and *p*[*n*] or *q*[*n*] as

(12) $$\begin{array}{c} S\left[n\right]=W^{\prime }\;p\left[n\right]. \end{array}$$

Thus, it is observed that standard CCA algorithm uses the matrix inverse operation to solve the linear equations by obtaining the maximum value of *ρ*^2^ for determining the demixing matrix *W*.

In this research paper, a fast and efficient modified CCA algorithm is proposed which uses the backslash or left matrix division operator for solving the linear (10) and (11) to calculate Eigen values. This backslash operator is fast and efficient than the inverse operator. The remaining algorithm is similar to the CCA approach. Let us define the linear (10) as

(13) $$\begin{array}{c} G=\rho^{2}=inv\left(C_{xx}\right)*C_{xy}*inv\left(C_{yy}\right)*C_{yx}, \end{array}$$

(14) $$\begin{array}{c} G=\rho^{2}=A*B, \end{array}$$

where

(15) $$\begin{array}{c} \;A=inv\left(C_{xx}\right)*C_{xy}={C_{xx}}^{-1}*C_{xy}, \end{array}$$

(16) $$\begin{array}{c} B=inv\left(C_{yy}\right)*C_{yx}={C_{yy}}^{-1}*C_{yx}. \end{array}$$

The parameters *A* and *B* from linear (15) and (16) are defined as

(17) $$\begin{array}{c} \begin{array}{l} A*Cxx=Cxy,\\ B*Cyy=Cyx. \end{array} \end{array}$$

Standard CCA method uses matrix inverse to obtain the value of *A* and *B*. In this paper, the left matrix division is proposed to solve these linear (15) and (16).

The left division operator as *Cxx*\*Cxy* can be defined as a matrix division of *Cxx* into *Cxy*, which is equal to the solution of inv(*Cxx*)∗*Cxy*. If *Cxx* is an *N*-by-*N* matrix and *Cxy* is a column vector with *N* components or a matrix containing several such columns, then *A*∗*Cxx* = *Cxy* equation can be defined in terms of left division operator as *A* = *Cxx*\*Cxy*.

The similar solution can be defined for the matrix *B*. The solution of (15) and (16) can be written as

(18) $$\begin{array}{c} A=Cxx\backslash Cxy, \end{array}$$

(19) $$\begin{array}{c} B=Cyy\backslash Cyx. \end{array}$$

Equations (18) and (19) suggest that the role of inverse operation is avoided to obtain the value of parameters *A* and *B*. This will simplify the equation and thus computation time too.

#### 2.2.1. The Justification of Left Division Operator Employed in GECCA

In order to prove left division operator, the numerator and denominator are multiplied by numerator transposed as

(20) $$\begin{array}{c} A={Cxx}^{T}*Cxx\backslash {Cxx}^{T}*Cxy,\\ B={Cyy}^{T}*Cyy\backslash {Cyy}^{T}*Cyx. \end{array}$$

By shuffling the order of operator by using Gaussian elimination concept,

(21) $$\begin{array}{c} A={Cxx}^{T}*\left(Cxx*{Cxx}^{T}\right)\backslash Cxy, \end{array}$$

(22) $$\begin{array}{c} B={Cyy}^{T}*\left(Cyy*{Cyy}^{T}\right)\backslash Cyx, \end{array}$$

since *Cxx* and *Cyy* are *N*-by-*N* symmetric matrix and *Cxy* and *Cyx* are column vectors with *N* components or a matrix with several such columns.

Since *Cxx*∗*Cxx*^T^ = *Cyy*∗*Cyy*^T^ = 1, therefore, (21) and (22) can be written as

(23) $$\begin{array}{c} A={Cxx}^{T}*\left(1\right)\backslash Cxy, \end{array}$$

(24) $$\begin{array}{c} B={Cyy}^{T}*\left(1\right)\backslash Cyx. \end{array}$$

For symmetric and square matrix, *Cxx*^*T*^ = *Cxx* and *Cyy*^*T*^ = *Cyy*.

Then (23) and (24) are simplified as

(25) $$\begin{array}{c} A=Cxx\backslash Cxy, \end{array}$$

(26) $$\begin{array}{c} B=Cyy\backslash Cyx. \end{array}$$

Replacing the values of *A* and *B* from (25) and (26) to (14), the solution of *G* is obtained as

(27) $$\begin{array}{c} G=\left(Cxx\backslash Cxy\right)*\left(Cyy\backslash Cyx\right). \end{array}$$

The value of *G* is obtained without inverse operation by just applying left division operator. This solution not only saves the computation time but also increases the efficiency of the CCA method with minimizing the error. The obtained G = *ρ*^2^ is the Eigen value with *k* in descending order, that is, *k*_1_ > *k*_2_ > *k*_*n*_; *a* and *b* are the Eigen vector for the greatest value of *k*.

Then the CCA component *p*[*n*] = [*p*_1_, *p*_2_,…, *p*_*n*_] are obtained by placing the vector *X*[*n*] in (8), and sources are expected by using the weighted demixing matrix from *X* by using (12).

If *A* is a *M* × *N* matrix with *M* ≤ *N* and *B* is a column vector with *M* components, or a matrix with several such columns, then *X* = *A*\*B* is the solution in the least squares sense to the under or over determined system of equations *A*∗*X* = *B*. The effective rank *K* of *A* is determined from the QR decomposition with pivoting. The solution *X* is computed which has at most *K* nonzero components per column.

#### 2.2.2. Benefits of GECCA over CCA Algorithm

The advantage of GECCA Algorithm over CCA are as follows:

1. The operation of CCA algorithm depends on the inverse operation to find the demixing matrix. If the input matrix is not a square matrix, then this CCA algorithm fails. However, left division operator-based GECCA algorithm provides a solution if input matrix is not square.
2. The CCA algorithm involves the inverse of the matrix for their operation. The inverse operation is computationally complex and time-consuming. However, proposed GECCA algorithm is faster than CCA for obtaining the correlation-based source separation by employing left division operator in place of inverse operation.
3. The proposed GECCA algorithm employs left division operator. This operator allows better estimates of the matrix inversion. These efficiently estimated coefficients once applied to SWT filter improve the SWT filtering efficiency thus improving the overall efficiency of the motion artifact removal algorithm.
4. The CCA algorithm operation is not consistent in each situation. This issue can be overcome by the proposed GECCA algorithm.

The above discussed efficient and fast correlation-based algorithm applied with WT algorithm is discussed in the subsequent section.

### 2.3. Wavelet Transform (WT)

The artifacts available in the EEG signal are suppressed with cascaded approach of EEMD and GECCA. Although, some brain actions may be disturbed due to high-frequency sensor noise and low-pass and high-pass filter components. As these noise signal frequencies will get overlap with the brain signals, then conventional filtering technique cannot be utilized and thus this WT is applied to take away unwanted noises from EEG signal [12].

The most frequently applied wavelet transform is discrete wavelet transform (DWT). In the case of EEG artifact removal, preserving neural information of the signal is determinant. Thus, some latest research shows that stationary wavelet transform (SWT) is a powerful tool to remove artifacts of the signal with preserving the neural information of original EEG signal [14].

SWT algorithm is translation invariant, so no downsampling of the data is involved. Translation-invariance is achieved by removing the down- and upsamplers as in the DWT and upsampling the filter coefficients by a factor of 2^(*j* − 1)^ at the *j*th level of the algorithm. SWT is preferred as it removes unpredictable behavior and noise randomness in the EEG signal due to the motion artifacts remaining after two-stage filtering of EEG signals. Therefore, EEG signal gets smoothened over the length with containing all their fundamental properties. Finally, proposed algorithm based on discussed algorithm is discussed in the next section.

## 3. Proposed Algorithm

The proposed artifact removal algorithm is as follows:

1. Define the reference EEG data from the multichannel data set for correlation match.
2. The input artifact EEG channel is passed through EEMD to decompose with 3 ensembles to convert single-channel EEG into multichannel EEG data.
3. Generated IMFs are passed to GECCA algorithm for source separation.
4. The GECCA output contains traces of artifacts in the form of randomness and noise. Thus, the output of GECCA is applied further to SWT algorithm for effective artifact removal. The decomposition through SWT is performed on the frequency domain of the signal with Rigrsure thresholding.
5. Pearson's correlation coefficient is used for artifact recognition and their suppression and finally reconstructed to find the artifact-free signal.
6. Parametric evolution is carried out based on SNR and correlation improvement. The correlation is carried out with respect to reference original EEG data initially defined.
7. The parametric evaluation of the proposed method is done with existing motion artifact removal methods.

## 4. EEG Signal Data Set

**Figure 1:**
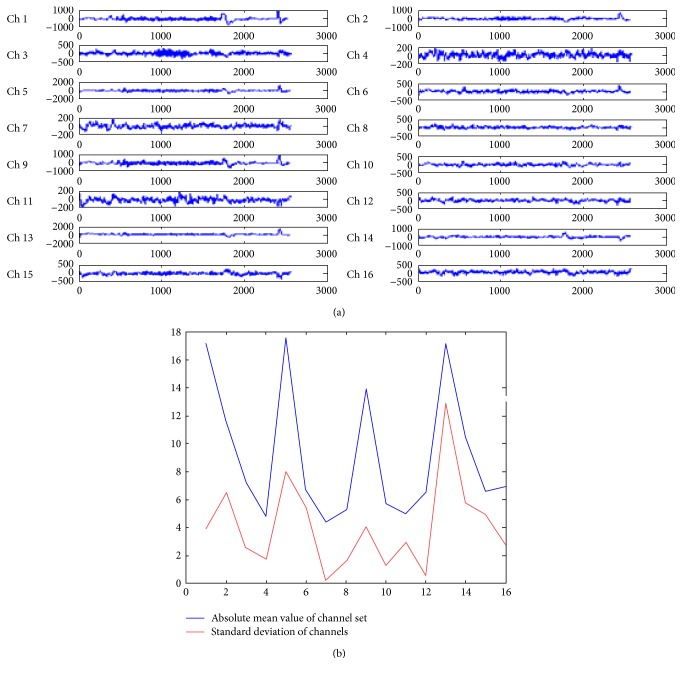
(a) Multichannel EEG data set containing the muscular and eye blink motions. (b) Statistical properties of EEG channels.

The EEG data is a multichannel data considered from standard MIT scalp data set with 24 channels recorded with different electrodes. This dataset is available on PhysioNet. The data is taken for 10 seconds and with 2560 samples/signals. Sampling frequency is taken as 256 Hz, and sampling interval is of 0.00390625 sec. These data samples are reduced to 16 channels to satisfy the international standard of 10–20 channels.  Figure 1() shows the first 16 channels of EEG data set available online with chb01_01_edfm.mat available at PhysioNet provided by Shoeb [15]. It is observed from Figure 1() that each channel is recorded with different electrode having different motion characteristics.

In Figure 1(a), the channels 1, 5, 9, and 13 correspond to the eye blinks as shown by negative high peaks (scale of 1000 and 2000). This was displayed and referenced by Shoeb [15]. These channels (1, 5, 9, and 13) have maximum motion or artifacts since they have higher standard deviation 172.0907, 175.417, 136.129, and 170.467, respectively. In this paper, channel numbers 4, 7, and 11 have the least artifact effects (only with scale of 200) out of the 16 channel EEG signal, this can be quantitatively justified by their lowest mean value and standard deviation (on division by 10 scale) as shown in Figure 1(b). The channel 7 in Figure 1(a) has the smallest standard deviation (=4,364,903) and the smallest absolute mean value (=0.228125) as shown in Figure 1(b) which justifies the minimum motion artifacts. Thus, the channel number 7 is used as a reference signal for correlation-based artifact removal. This reference signal is termed as original signal in the paper.

**Figure 2:**
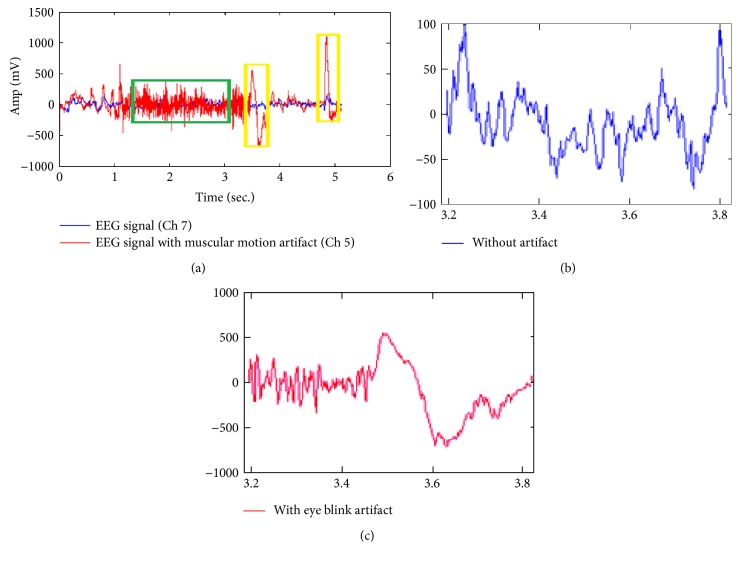
(a) Comparison of referenced channel 7 and artifact channel 5 for first 10 seconds. (b) Enhanced reference data channel 7. (c) Artifact channel 5 for 3-4 seconds respective to yellow rectangle.

Figure 2 compares the original or reference EEG data (channel number 7) with blue color and artifactual signals (channel number 5) with red color. The channel 5 is shown here for the comparison because it has maximum standard deviation of 175.417 with maximum peak variations with eye blink. It is observed from Figure 2 that raw EEG data may suffer from either muscular motion artifact (shown under green rectangle) or eye blink artifact (shown under yellow rectangle) or both of them. The eye blink artifact may cause amplitude increase about 10 times greater than the original EEG data. The EOG artifact overlaps with EEG signal in impulsive form, while muscular motions cause random broad spectrum amplitude variations. Nature of the reference EEG data with and without artifacts is clearly shown in Figures 2(b) and 2(c).

The eye blink artifact may cause amplitude increase about 10 times greater than the original EEG data. The EOG artifact overlaps with EEG signal in impulsive form, while muscular motions cause random broad spectrum amplitude variations. In this paper, the muscular motion artifact signals (as in channel 5) are naturally generated at the time of EEG data capturing with electrodes. Therefore, for the sake of clarity, the enhanced version of reference and artifact signal is shown in Figures 2(b) and 2(c).

## 5. Result and Discussion

**Figure 3:**
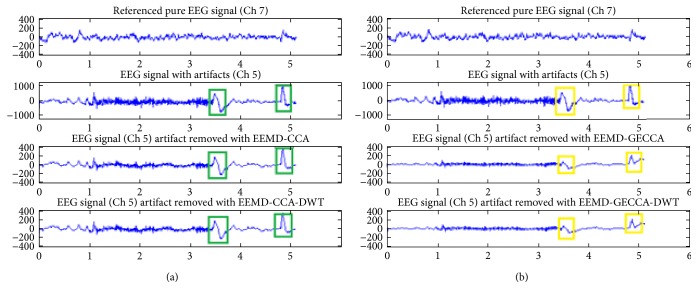
Comparisons of EEG artifact removal methods (a) with CCA and DWT and (b) with GECCA and DWT.

The novel GECCA artifact removal approach is evaluated on artifactual EEG channel number 5 and compared with CCA algorithm. Comparison of the EEG artifact removal methods for channel number 5 using CCA and GECCA along with DWT is shown in Figure 3. The EEG signal channel number 7 is considered as pure EEG signal. The comparison of the green and yellow boxes in Figures 3(a) and 3(b) suggests that blind source separation- (BSS-) based proposed GECCA method removes the motion artifacts significantly better than the existing CCA-based method. Green rectangles are correspondent to CCA, and yellow rectangles are correspondent to GECCA. It is observed that the eye blink peaks with GECCA-DWT are minimized to half that of the eye blink peaks with CCA-DWT (of the order of ±200 is reduced to around ±100) as mentioned in fourth row of Figure 3. Moreover, the pattern of the second blink is also much similar to the reference EEG signal after GECCA-DWT.

**Figure 4:**
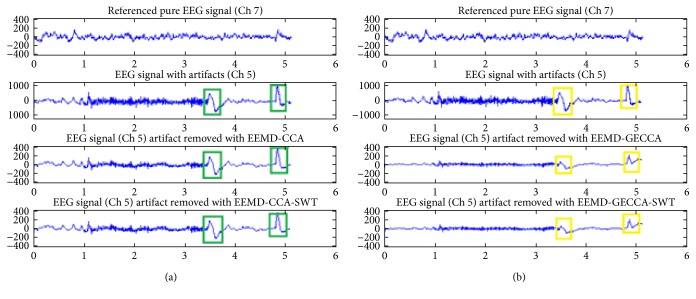
Comparisons of EEG artifact removal methods (a) with CCA and SWT and (b) with GECCA and SWT.

The SWT algorithm is more proficient for artifact removal of neural EEG signal in comparison to DWT algorithm; consequently, the efficacy of proposed BSS approach (GECCA) is also evaluated with SWT and compared with CCA-based approaches.  Figure 4 provides the comparison of the EEG artifact removal methods using CCA and GECCA along with SWT filtering. The artifact removal approaches are applied on channel number 5, and performance is compared with reference channel 7 EEG signal. The comparison of the respective green and yellow boxes in Figures 4(a) and 4(b) suggests that the proposed GECCA method removes the motion artifacts significantly better than the existing CCA-based method. In addition, the vigilant observation and comparison of the respective green rectangles in Figure 4(a) and yellow rectangles in Figure 4(b) suggests that GECCA with SWT removes the eye blink effect better than CCA-SWT. It is observed that the eye blink peaks with GECCA-SWT are minimized to half that of the eye blink peaks with CCA-SWT (of the order of ±200 is reduced to around ±100) as mentioned in the fourth row of Figure 4. This could be quantitatively even better clear in the next sections with evaluation parametric comparison encapsulated in Table 1.

**Figure 5:**
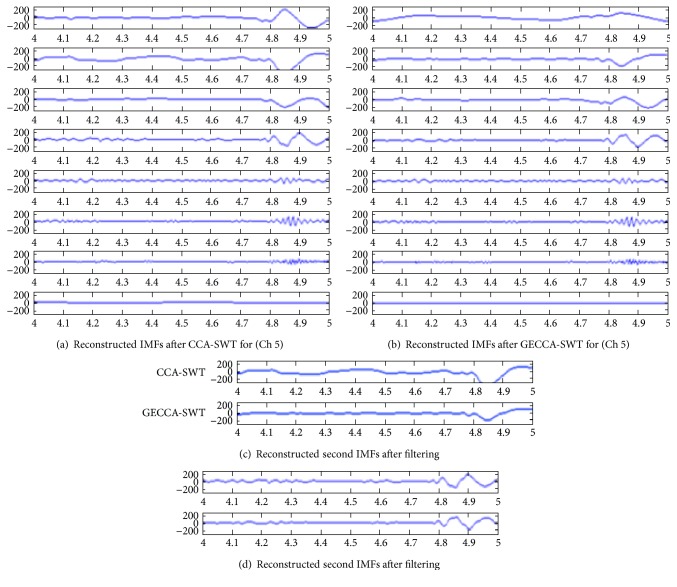
Reconstructed IMFs after (a) CCA-SWT, (b) GECCA-SWT, and (c) comparison of CCA and GECCA for the 2nd and 4th reconstructed EEMD (IMFs).

The 8 reconstructed signals after SWT-smoothened approach are compared for CCA and GECCA as shown in Figure 5. It is observed that GECCA method not only preserves the information but also provides better and fast estimates. This is concluded by comparing the 2nd and 4th frequency bands as shown in Figures 5(c) and 5(d).

**Figure 6:**
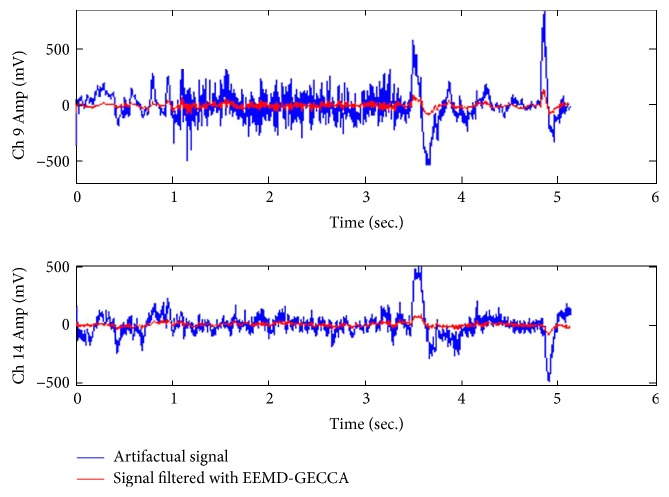
Artifact removal consequences with the proposed (EEMD-GECCA-SWT) method.

Moreover, the proposed artifact removal method (EEMD-GECCA-SWT) outputs are compared for three distinct EEG channel data, namely, Ch 9, Ch 4, and Ch 14, as presented in Figure 6. In Figure 6, blue color presents the original artifactual signal, whereas red color signal represents the filtered output by the proposed approach. It is demonstrated that proposed method of GECCA removes the eye blink artifacts and motion artifacts significantly.

In order to evaluate the performance of the proposed method, parametric evaluation is carried out as shown in Table 1. The performance of artifact removal methods is carried out for CCA and GECCA methods along with DWT and SWT filtering methods. It is observed that proposed method EEMD-GECCA-SWT reduces the mean square error (MSE) by around 15.81% and improves the DSNR performance significantly. Moreover, the higher lambda (*λ*) value indicates the improved filtering performance [8]. Table 1 shows that GECCA-based artifact removal methods attain improved lambda (*λ*) value. The vigilant observation of results on Table 1 also presents better correlation improvement of GECCA when compared to CCA, both for DWT and SWT. The backslash operator provides better estimation efficiency. Thus, Gaussian elimination-based approach provides improved correlation by using backslash operation which is more efficient than the matrix inverse method.

The proposed method evaluation is also carried out with the receiver operation characteristic (ROC) curve parameters as shown in Table 2. The positive predictive value (PPV), negative predictive value (NPV), sensitivity (Sen), and specificity (Spe) are calculated by true positive (TP), true negative (TN), false positive (FP), and false negative (FN) [16] as follows:

(28) $$\begin{array}{c} Sen=\frac{TP}{TP+FN}, \end{array}$$

(29) $$\begin{array}{c} Spe=\frac{TN}{TN+FP}, \end{array}$$

(30) $$\begin{array}{c} PPV=\frac{TP}{TP+FP},\; \end{array}$$

(31) $$\begin{array}{c} NPV=\frac{TN}{TN+FN}. \end{array}$$

The true positive (TP) indicates that the sample is identified as an artifact when it was actually an artifact. False positive (FP) presents that the sample is identified as an artifact but actually it was not. The false negative (FN) is an indicator for the absence of artifact; however, the artifact was present in the sample. Moreover, true negative (TN) indicates the absence of the artifact when the artifact was actually not present.

**Figure 7:**
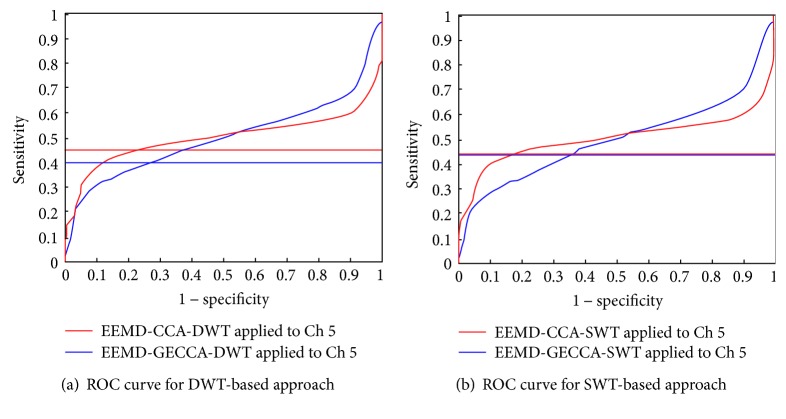
ROC plot comparison for CCA and GECCA (a) based on DWT and (b) based on SWT.

This ROC curve is a comparison plot between true positive rate or sensitivity and false positive rate (1 − specificity) [16] for accurate detection of artifact from EEG signal as shown in Figure 7().

Figure 7(a) shows comparison of CCA and GECCA approaches based on DWT. Blue and red color horizontal line indicates the sensitivity of EEMD-CCA-DWT and EEMD-GECCA-DWT artifact removal methods, respectively. The sensitivity or true artifact detection rate for CCA-based approach is 0.39961, whereas GECCA-based approach presents improved and fast response with increased value of 0.45156. However, Figure 7(b) shows SWT-based approach comparison for CCA and GECCA algorithm with the same blue and red color code. Both approaches present almost similar sensitivity 0.43438 and 0.43789, respectively, as shown in Table 2. Although, GECCA-based approach performs better than CCA artifact removal algorithm. Moreover, the sensitivity is associated with accuracy of the algorithm too. All the ROC parameters as sensitivity, specificity, PPV, and NPV as mentioned above in (28), (29), (30), and (31) are calculated for EEG signal channel number 5 and tabulated for effective comparison of CCA and GECCA-based approaches as shown in Table 2. It is observed that GECCA-based approach shows improved sensitivity and thus better accuracy than CCA-based approaches with both DWT and SWT filtering. Moreover, specificity, PPV, and NPV also improve by GECCA-based methods.

In addition, the efficiency of GECCA-based artifact removal approach is also evaluated by considering ROC parameters for most artifact contaminated channel numbers (1, 3, 5, 6, 9, and 14) from Figure 1.  Table 3 encapsulates all the ROC parameters for filtering approach based on CCA and GECCA. It is observed from Table 3 that GECCA-based methods show improved accuracy in comparison to CCA-based method except channel 1. Similarly, specificity, PPV, and NPV parameters are also improved for most of the channel which presents the success of GECCA-based artifact removal approach.

The use of the left matrix division operator in the GECCA algorithms executes faster than the inverse matrix method used in CCA algorithm. Therefore, the proposed GECCA method is faster than the CCA. Moreover, left matrix division allows better estimates of the matrix inversion which improves the SWT filtering efficiency thus improves the overall efficiency of the Pearson's correlation-based artifact removal.

**Figure 8:**
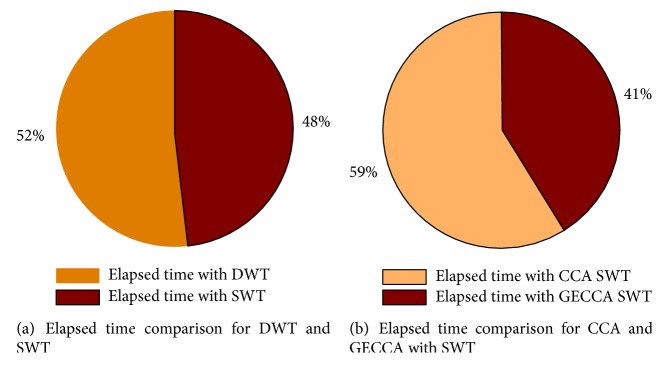
(a) Elapsed time comparison for DWT and SWT approach and (b) elapsed time comparison for proposed GECCA method with CCA method.

This execution or computation speed is evaluated by elapsed time. The elapsed time comparison of DWT and SWT is shown in Figure 8(a) suggesting that SWT is faster than DWT.  Figure 8(b) compares the elapsed time for GECCA and CCA with SWT. This presents that GECCA with SWT algorithm computation time is faster than CCA with SWT approach.

Figure 8(a) suggests that SWT is faster than DWT approach for artifact removal. Thus, SWT elapsed time is evaluated with CCA and GECCA approaches and concluded that GECCA approach with SWT is faster and efficient for EEG artifact removal.

## 6. Conclusion

The measurement and processing of EEG signal result in the probability of signal contamination prominently through motion artifacts which can obstruct the important features and information quality existing in the original EEG signal. To diagnose the human neurological diseases like epilepsy, tumors, and problems associated with trauma [15], these artifacts must be properly pruned assuring that there is no loss of the main attributes of EEG signals. Thus, a novel algorithm GECCA is introduced in cascade with EEMD and SWT for fast and effective suppression of motion artifacts from single-channel EEG signal. The proposed GECCA method uses backslash operation to solve the linear equations. This improves the computation efficiency of the methods. The application of SWT instead of DWT improves the SNR performance of the method and faster than the DWT method too. However, the proposed algorithm may give over smoothening if not properly designed. The proposed method based on GECCA is 18% faster than the conventional CCA. The various evaluation parameters as del signal to noise ratio (DSNR), lambda (*λ*), root mean square error (RMSE), and ROC parameters are employed to compare the performance of the proposed artifact removal algorithm. The ROC parameter comparison suggested that the improved accuracy is attained with GECCA algorithm except for Ch 1. The optimum evaluation result shows the success of proposed motion artifact removal method.

## Figures and Tables

**Table 1:** Parametric comparison of the artifact removal methods with CCA and GECCA.

Algorithm	With DWT filtering		With SWT filtering	
Parameters	EEMD-CCA	EEMD-GECCA	EEMD-CCA	EEMD-GECCA
MSE	69.3283	51.2272	70.2484	50.1366
Correlation improvement	0.0019	0.0666	0.0103	0.0654
Lambda	66.8838	86.0016	66.2544	87.2759
DSNR	17.7248	29.0387	17.2621	30.2080

**Table 2:** Comparison of the ROC parameters with CCA and GECCA for channel 5.

Algorithm	DWT filtering with		SWT filtering with	
Parameters	EEMD_CCA	EEMD_GECCA	EEMD_CCA	EEMD_GECCA
Sensitivity	0.39961	0.45156	0.43438	0.43789
Specificity	0.74062	0.78477	0.65469	0.82656
Accuracy	57.0117%	61.8164%	54.4531%	63.2227%
PPV	60.6402%	67.7211%	55.7114%	71.6294%
NPV	55.2287%	58.8632%	53.6492%	59.5218%

**Table 3:** Comparison of ROC parameters for 6 different EEG channels for proposed method.

Method ↓	Parameters in %	Ch 1	Ch 3	Ch 5	Ch 6	Ch 9	Ch 14
With GECCA	Accuracy	57.3047	59.4141	63.2227	65.3125	63.0859	63.5352
	PPV	57.18	57.0674	71.6294	75.0319	71.9241	59.802
	NPV	55.3368	64.0936	59.5218	61.0298	59.3263	71.8612
With CCA	Accuracy	57.6953	58.8672	54.4531	60.2734	62.2461	61.2109
	PPV	56.0578	62.6251	55.7114	64.7422	69.3399	58.2566
	NPV	60.546	56.8332	53.6492	57.8837	58.9597	67.4574
